# Restoration of missing regions in limited field of view computer tomography using an image- and sinogram-based conditional generative adversarial network model

**DOI:** 10.1093/bjrai/ubag010

**Published:** 2026-05-04

**Authors:** Hideaki Hirashima, Kohei Arimoto, Takao Onishi, Megumi Nakao, Takashi Mizowaki, Mitsuhiro Nakamura

**Affiliations:** Department of Radiation Oncology and Image-Applied Therapy, Graduate School of Medicine, Kyoto University, Sakyo-ku, Kyoto 606-8507, Japan; Department of Advanced Medical Physics, Graduate School of Medicine, Kyoto University, Sakyo-ku, Kyoto 606-8507, Japan; Department of Advanced Medical Physics, Graduate School of Medicine, Kyoto University, Sakyo-ku, Kyoto 606-8507, Japan; Department of Biomedical Engineering and Intelligence, Graduate School of Medicine, Kyoto University, Sakyo-ku, Kyoto 606-8507, Japan; Department of Radiation Oncology and Image-Applied Therapy, Graduate School of Medicine, Kyoto University, Sakyo-ku, Kyoto 606-8507, Japan; Department of Advanced Medical Physics, Graduate School of Medicine, Kyoto University, Sakyo-ku, Kyoto 606-8507, Japan

**Keywords:** limited FOV, restoring missing region, deep learning, sinogram completion, CBCT

## Abstract

**Objectives:**

This study aimed to restore missing regions from the limited field of view (FOV) using image- and sinogram-based conditional GAN (cGAN) models.

**Methods:**

cGANs are deep learning frameworks that generate realistic data via a competitive neural network process. We used planning CT (pCT) datasets from 96 patients: 64 for training, 16 for validation, and 16 for internal testing. Two cGAN models (image-based and sinogram-based) were developed to generate body contour outside the FOV. Next, 23 cone-beam CT (CBCT) datasets were evaluated as an external test group.

**Results:**

In pCT internal test datasets, the median values for mean absolute error (MAE), root mean square error (RMSE), and structural similarity index measure (SSIM) for each model were as follows: image-based model—101.73 HU for MAE, 39.26 HU for RMSE, and 0.83 for SSIM; sinogram-based model—16.91 HU for MAE, 23.19 HU for RMSE, and 0.91 for SSIM. In CBCT external test datasets, the sinogram-based model outperformed the image-based model with a median MAE of 73.32 HU versus 180.72 HU, a median RMSE of 37.02 HU versus 43.42 HU, and a median SSIM of 0.75 versus 0.63. The sinogram-based model demonstrated significant improvements in MAE, RMSE, and SSIM (*P *< .05).

**Conclusions:**

The sinogram-based cGAN model exhibits considerable potential for restoring missing regions outside the FOV, outperforming the image-based model in accuracy metrics.

**Advances in knowledge:**

This model offers a novel approach to accurately predict missing regions from a limited FOV, enhancing continuity of the body contour while accommodating patient-specific variations.

## Introduction

On-board kilovoltage cone-beam CT (CBCT) is a crucial imaging modality in image-guided radiation therapy, providing essential anatomical information for precise patient positioning, monitoring the daily shape and position of organs, and dose calculation.^1^

CBCT offers 2 scan modes: half-fan and full-fan. The half-fan mode is used for imaging larger areas, such as the body, by covering only part of the detector. While this provides a wider field of view (FOV) and captures more anatomical structures, it requires slightly longer imaging times and higher radiation doses. In contrast, the full-fan mode is typically used in clinical practice when the target is small, and full-body visualization is not necessary for alignment. In this mode, the x-ray beam covers the entire detector, resulting in shorter imaging times and lower radiation doses compared with the half-fan mode. Its short imaging time makes it useful for breath-hold radiotherapy.^2^^,^^3^ However, the full-fan mode commonly results in missing anatomical structures outside the FOV. These limitations can lead to inaccuracies in dose calculations during post-analysis if changes in the shape and position of organs outside the FOV are not accurately considered.

To address these problems, studies have focused on reducing artifacts and enhancing dose calculation accuracy while preserving the advantages of the full-fan mode.^3–5^ These studies have developed techniques to monitor changes in the shape of organs within the FOV and improve dose calculation accuracy using limited FOV CBCT images. The results of studies indicate that deriving images similar to those obtained with half-fan mode from limited FOV CBCT could facilitate accurate organ positioning and dose calculation.^3–5^ However, limited studies have focused on the restoration of missing areas from a limited FOV.

Conventional image processing approaches for restoring missing regions from limited FOV include integrating limited CBCT data with original CT scans or using deformable image registration techniques to map limited FOV CBCT images onto anatomical CT structures.^6^ Addressing patient-specific variations is challenging, which renders the restoration of missing regions challenging. Deep learning (DL) methods, such as convolutional neural networks (CNNs) and generative adversarial networks (GANs), have been used to address this problem.^7–12^ Conceptually, GANs consist of 2 competing models: a generator that attempts to create realistic synthetic data (eg, missing anatomy) and a discriminator that evaluates whether the generated data is real or fake. This adversarial training allows the model to produce highly realistic and anatomically plausible images, making it well suited for medical image restoration. DL methods can be broadly categorized into 2 types, namely image-to-image translation^7–9^ and projection-to-projection translation.^10–12^

Image-to-image translation methods use CNNs and GANs to learn directly from the original images and use relationships between image datasets to restore missing regions. Advances in these models have improved their ability to capture comprehensive features and facilitate data restoration.^7–9^ By contrast, projection-to-projection translation methods utilize GAN to restore missing regions from projection data, such as sinograms, preserving the integrity of the projection data while addressing the limited FOV problem.^10–12^ Sinogram completion, which is a technique in CT or CBCT used to fill in missing data in sinograms, can not only restore body contour but also address problems such as truncation artifacts and cupping artifacts.^10–12^ However, the efficacy of both image-to-image translation and projection-to-projection translation methods in restoring missing regions from a limited FOV is yet to be thoroughly investigated, particularly in terms of their effectiveness across various CT reconstruction methods.

This study developed a DL model to restore missing regions from a limited FOV and evaluate the accuracy of both image- and sinogram-based models using planning CT (pCT) data. The reason for using pCT data is the insufficient number of CBCT images containing complete anatomical information within the FOV in our institution; therefore, we opted to train the model using pCT data, which offers a larger sample size and comprehensive anatomical coverage, and then evaluated the performance of the model on real CBCTs.

## Methods

### Workflow

**Figure 1:**
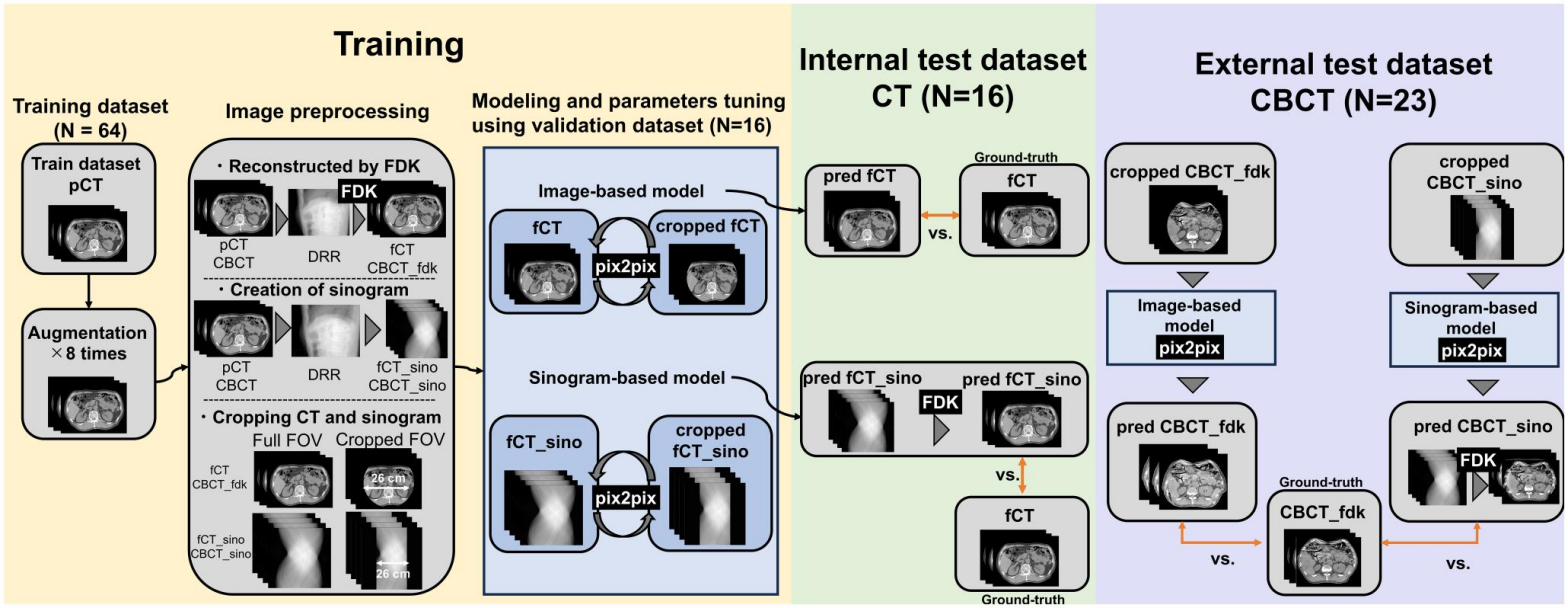
Workflow for restoring missing regions from a limited field of view using image- and sinogram-based model.

The workflow of this study was organized into 4 distinct steps: (1) data separation and augmentation within the training datasets; (2) image preprocessing, including the reconstruction of CBCT using digitally reconstructed radiographs (DRRs) from pCT, creation of sinograms, and image cropping; (3) development of both image- and sinogram-based models; and (4) evaluation of the models (Figure 1).

### Patient datasets

This study included 100 patients with pancreatic cancer who were treated with intensity-modulated radiation therapy. Ninety-six of these patients underwent single pCT, which was acquired under breath-hold conditions at the end of exhalation using a SOMATOM Definition scanner (Siemens Medical Systems, Erlangen, Germany). The pCT data from 96 patients were randomly divided into 3 subsets: 64 for training, 16 for validation, and 16 for internal testing. The FOV, pixel sizes, and slice thickness were 500, 0.98, and 2 mm for pCT, respectively. Data augmentation for the pCT datasets was applied during training, which expanded the original dataset by a factor of 8, resulting in training on 8-fold augmented datasets.

Additionally, the CBCT data, 3, 5, 5, and 10 CBCTs from 4 patients, were designated as external testing data to evaluate the performance of the developed model. CBCT datasets were obtained under breath-hold conditions at the end of exhalation using an Ethos system (Varian Medical Systems, Palo Alto, CA, USA), and inclusion criteria required a long-axis body thickness of >30 cm. The FOV, pixel sizes, and slice thickness were 500, 0.98, and 2 mm for CBCT, respectively.

Both pCT and CBCT data consisted of 512 × 512 pixels and included 70 slices. This study was approved by the Institutional Review Board under protocol number R1446-3.

### Image preprocessing

#### Reconstruction of CT and CBCT using the Feldkamp-Davis-Kress method

In this study, the ground-truth CT and CBCT images differed from those acquired in clinical practice. Although iterative reconstruction is widely used for clinical pCT and CBCT, this study used the Feldkamp-Davis-Kress (FDK) reconstructed images as ground truth, referred to as FDK-pCT (fCT) and FDK-CBCT (CBCT_fdk). The fCT and CBCT_fdk were reconstructed using the FDK method with DRRs from both pCT and CBCT. Owing to the limitations of the equipment, actual projection data could not be used. Therefore, DRRs were generated from both CT and CBCT data as a proxy and utilized as projection data for sinogram reconstruction.

#### Creation of sinogram image

In this study, sinogram completion was achieved by generating pseudo-projected images of DRRs from both pCT and CBCT. These DRRs were subsequently used to create sinogram images (fCT_sino and CBCT_sino), ensuring the accurate reproduction of all body contours. Each sinogram consisted of 2048 × 900 pixels and included 768 slices per patient. The sinogram settings were designed to simulate a typical C-arm linac (e.g., TrueBeam by Varian) full-mode CBCT, by using a detector size of 1024 × 768 pixels with the pixel size of 0.51 mm.

#### Cropping CT and sinogram image

For each fCT image, a circular FOV with a diameter of 26 cm was extracted. The cropped fCT image was subsequently padded with zeros around the edges, preserving only the inner 26-cm diameter region from the image’s center. This processing step ensured that the dimensions of the cropped image matched those of the full-fan mode used in a typical C-arm linac system.

### Conditional GAN model

Model building for image- and sinogram-based approaches was conducted using the pix2pix framework implemented in Python 3.9 and PyTorch 1.9, with training performed for up to 150 epochs. The pix2pix framework utilized conditional GAN technology to train generators for performing image-to-image transformations.^13^ Conditional GANs are particularly highly effective for image restoration because their adversarial training process enforces not only pixel-level accuracy but also high-frequency structural realism, which is crucial for producing anatomically correct images. Specifically, the pix2pix framework employs a U-Net architecture for the generator, which effectively captures both local details and global spatial context through skip connections. For the discriminator, it uses a PatchGAN architecture, which penalizes structural discrepancies at the scale of image patches, ensuring the generated regions blend seamlessly and realistically with the actual acquired data. The training process involved inputting cropped fCT (or cropped fCT_sino) images and corresponding ground truth fCT (or fCT_sino) images. The input image underwent a 16-bit-to-8-bit depth conversion. Subsequently, the model’s image normalization process scaled the pixel values to a range between 0 and 1. The loss function used in this study was designed to produce realistic and anatomically consistent reconstructions. This function incorporates 2 components, namely *L*_1_ loss and adversarial loss, encompassing both the generator and the discriminator.


(1) L1(G)=Ex,y[‖y-G(x)‖1],

where *x* represents the input cropped fCT, *y* denotes the ground-truth fCT, and *G*(*x*) signifies the generated output.


(2) Ladv(G,D)=Ex,y[log⁡D(x,y)]+Ex,y[log⁡(1-D(x,G(x)))],

where *D* denotes the discriminator network. The loss function is formulated as follows:


(3) L(G,D)=Ladv(G,D)+λL1(G),

where λ is a hyperparameter that functions as the weighting term for the task-dependent loss. The balance between the various loss components can be adjusted using this hyperparameter. In this study, λ was set to 100, based on a previous study.^13^ By combining *L*_1_ loss and adversarial loss, the generator is used to produce outputs that are both anatomically accurate (due to the *L*_1_ loss) and realistic (due to the adversarial loss). The final formulation is as follows:


(4) G*=argminG⁡maxD⁡L(G,D),

where *G* tries to minimize this objective against an adversarial *D* that maximizes it. This formulation addressed the challenges of generating plausible and structurally accurate CT and sinogram reconstructions, given the information loss inherent in the cropped inputs. The output data consisted of the predicted images (pred fCT and pred fCT_sino), which included predictions for the entire body. For evaluating the sinogram, 3D reconstructed images were generated from the predicted sinograms by using the FDK method.

Model training was performed on an HP Z8 G4 Workstation equipped with an Intel Xeon Gold 6136 CPU (3.00 GHz), an NVIDIA Quadro RTX 5000 GPU, and 12.0 GB of RAM.

### Model evaluation with fCT image

**Figure 2:**
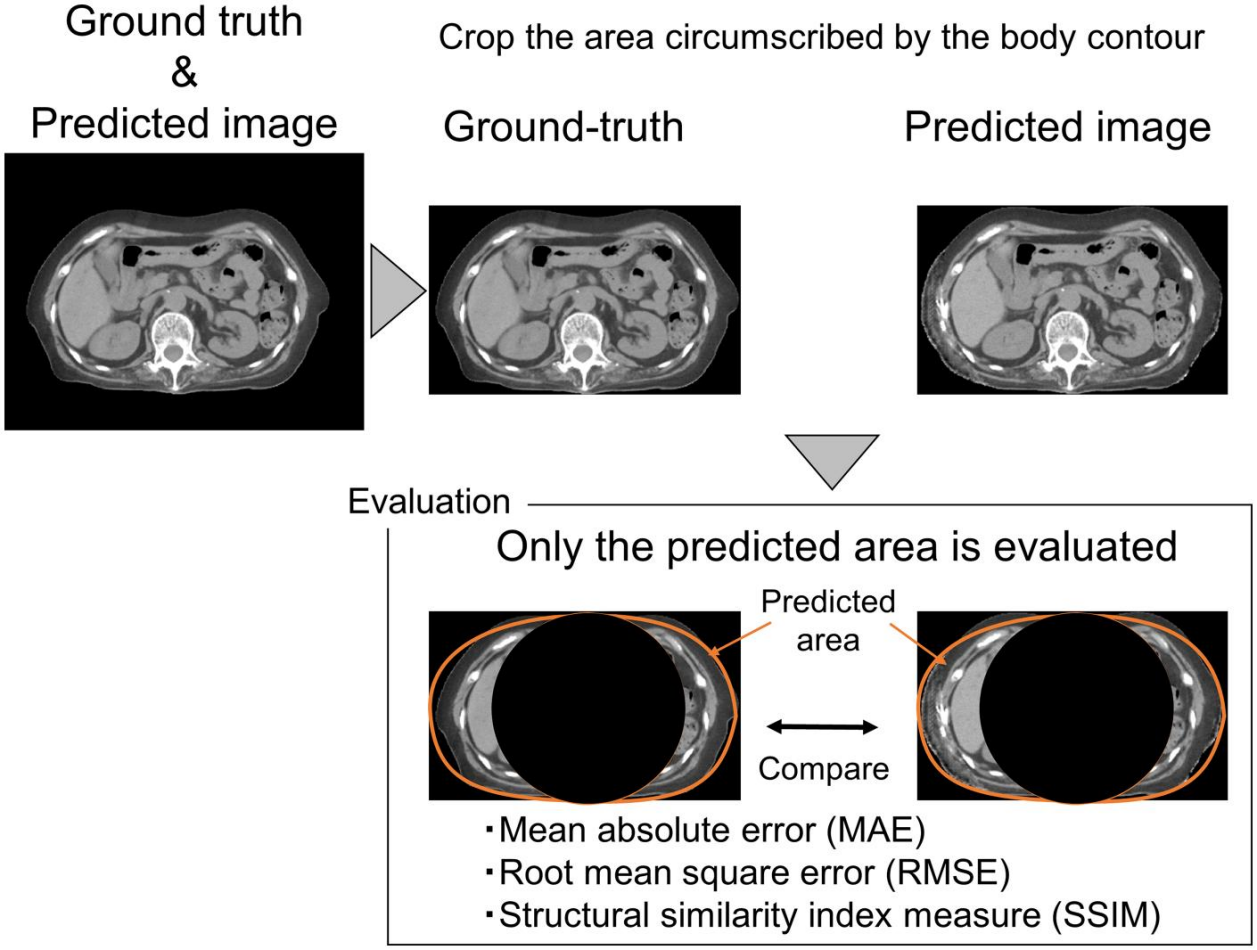
Evaluation metric between ground truth and predicted image.

In the evaluation, only the regions missing outside a 26-cm diameter from the center of the cross-section within the rectangular body defined by the body contour on the pCT were considered (Figure 2). Quantitative evaluation involved assessing the effectiveness of field expansion by comparing the predicted images obtained from both the image-based model and the sinogram-based model. The mean absolute error (MAE), root mean square error (RMSE), and structural similarity index measure (SSIM) were used for quantitative comparisons.


(5) MAE=1n∑i=1n|ai-fi|,

(6) RMSE=1n∑i=1n(ai-fi)2,

(7) SSIM= (2μaμf+C1) (2σaf+C2) (μa2+μf2+C1) (σa2+σf2+C2),

where *n* denotes the total number of target images, *i* represents the index of an image, *a* is the predicted image, *f* is ground truth, *μ* is the mean pixel value, *σ_af_* is the covariance, *σ* is the standard deviation, and *C*_1_ and *C*_2_ are constants expressed as (*K*_1_*L*)^2^ and (*K*_2_*L*)^2^, respectively. Here, *L* is the dynamic range of pixel values, *K*_1_ and *K*_2_ are constants set to 0.01 and 0.03, respectively, based on a prior study.^14^

### Model evaluation with CBCT_fdk image

To evaluate the applicability of the trained models to CBCT, 23 CBCT_fdk external datasets were used for evaluation. The evaluation region was identical to that used for the fCT assessment (Figure 2). Quantitative evaluation was performed using MAE, RMSE, and SSIM.

### Statistical analysis

A paired *t*-test was performed to assess the statistical significance of the evaluation measures between the image- and sinogram-based models, with a significance level set at *P *< .05.

## Results

### Model evaluation with fCT image

**Figure 3:**
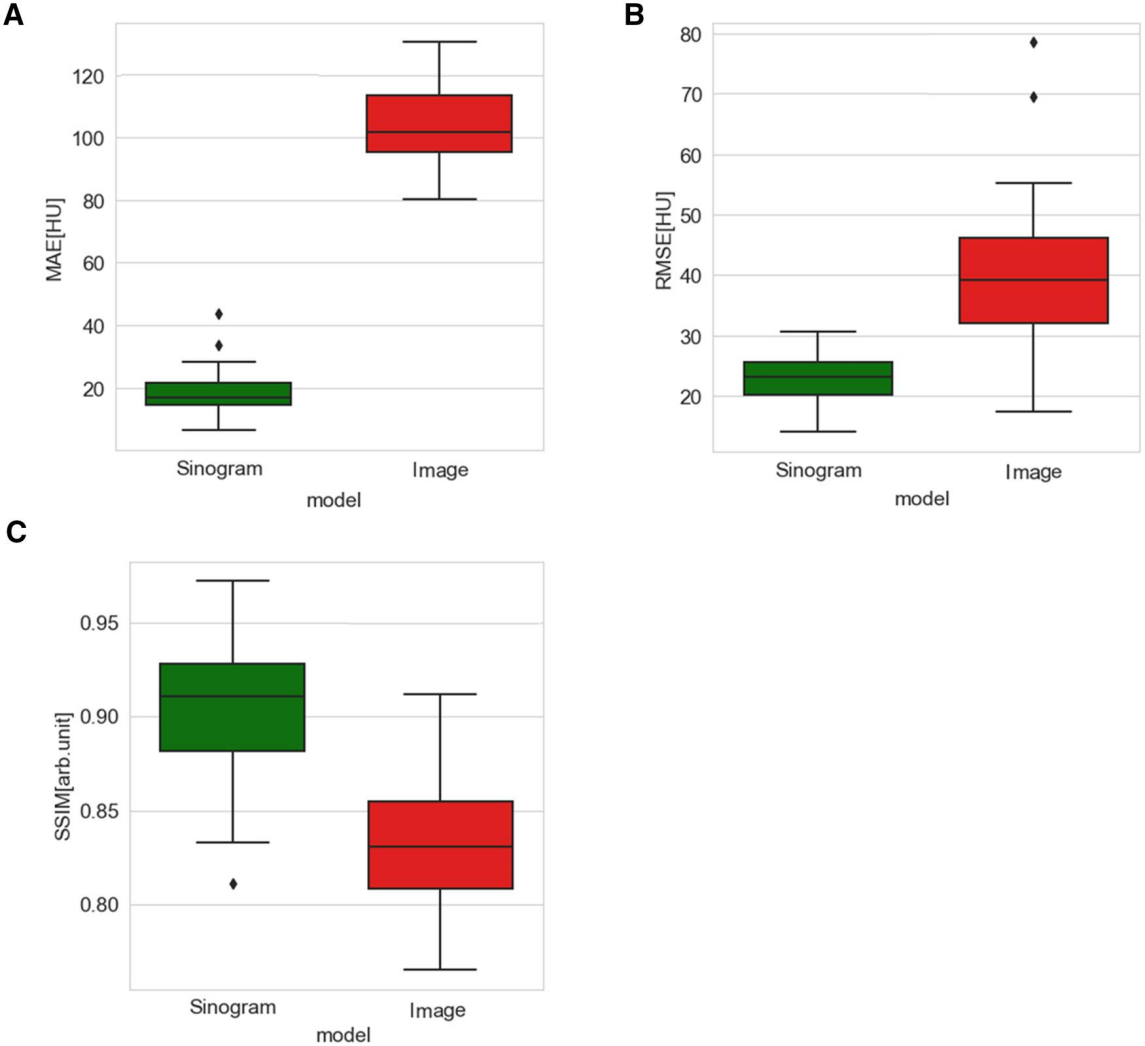
Box-and-whisker plots illustrating (A) mean absolute error (MAE), (B) root mean square error (RMSE), and (C) structural similarity index measure (SSIM) for the pCT internal test datasets. The green color denotes the sinogram-based model, while the red color denotes the image-based model.

The box-and-whisker plots presented in Figure 3 illustrate the distributions of the MAE, RMSE, and SSIM for the image- and sinogram-based models. The median values for MAE, RMSE, and SSIM were 101.73 HU, 39.26 HU, and 0.83 for the image-based model, and 16.91 HU, 23.19 HU, and 0.91 for the sinogram-based model, respectively. The MAE, RMSE, and SSIM for the sinogram-based model demonstrated significant improvements, compared with those for the image-based model (*P *< .05).

**Figure 4:**
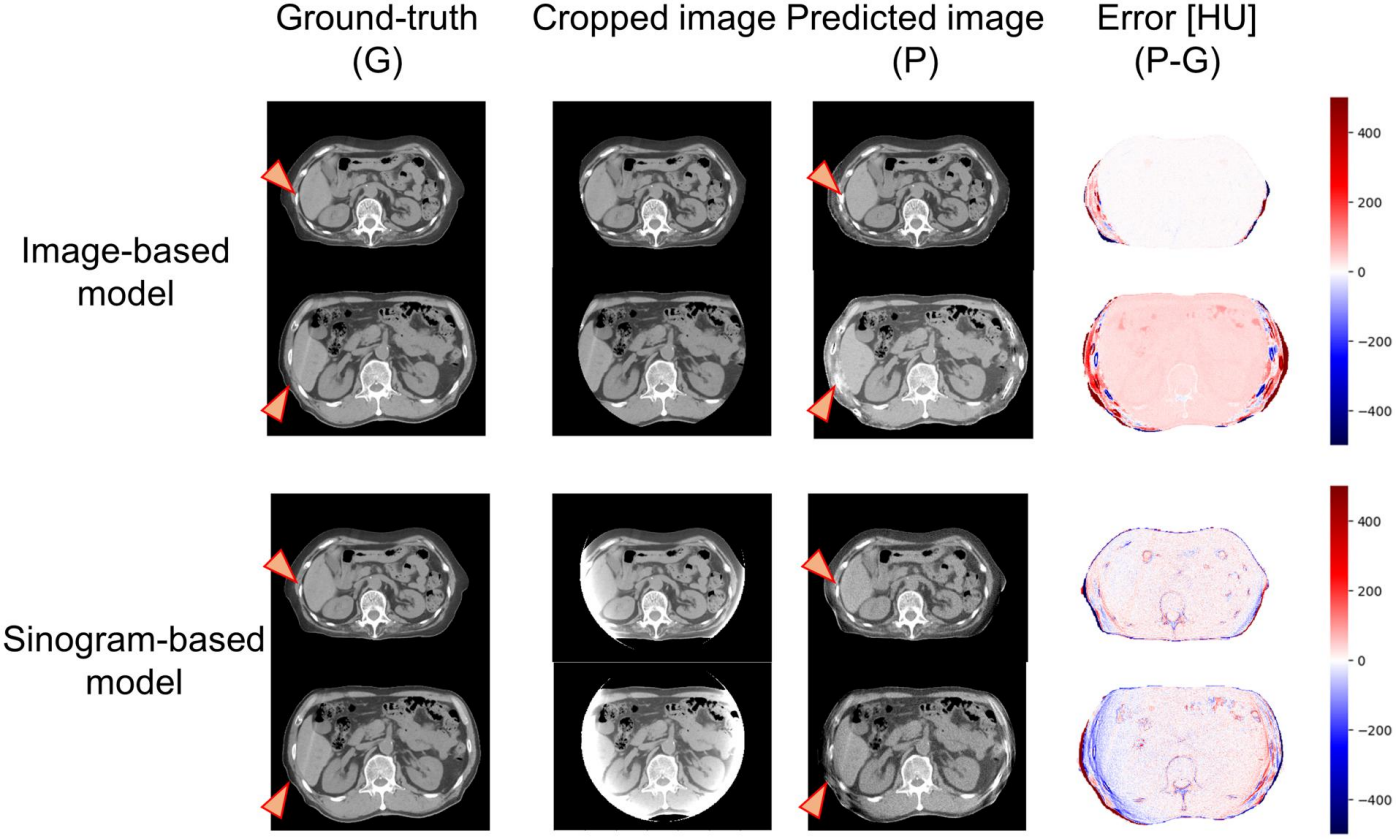
Representative patient images for the pCT internal test datasets, including ground-truth, cropped image, predicted image, and the difference (predicted image − ground-truth) for both image- and sinogram-based models. The window width was set to 400 HU, with the window level set to 0 HU. In the heatmap, the maximum value for red was set to 500 HU, and the maximum value for blue was set to −500 HU.

Figure 4 displays the cross-sectional heat maps of a representative patient, illustrating the differences between the predicted images and corresponding ground truth for the pCT internal test datasets. The heat maps reveal that the image-based models exhibit larger deviations in HU values outside body contours compared with the sinogram-based model. Notably, the sinogram-based model demonstrated highly accurate predictions of body contours, including patient-specific irregularities (as indicated by the arrows in Figure 4). By contrast, the image-based model exhibited misalignment in the predicted positions of the bones, resulting in substantial deviations in HU values relative to the sinogram-based model.

### Evaluation with CBCT_fdk image

The MAE, RMSE, and SSIM values were computed for the 23 CBCT_fdk datasets. The median values were 180.72 HU (range, 144.84-213.64 HU) for MAE, 43.42 HU (range, 31.35-57.07 HU) for RMSE, and 0.63 (range, 0.57-0.68) for SSIM in the image-based model. For the sinogram-based model, the median values were 73.32 HU (range, 45.69-108.95 HU) for MAE, 37.02 HU (range, 33.35-39.57 HU) for RMSE, and 0.75 (range, 0.68-0.83) for SSIM. The MAE, RMSE, and SSIM values for the sinogram-based model were significantly improved compared with those for the image-based model (*P *< .05).

**Figure 5:**
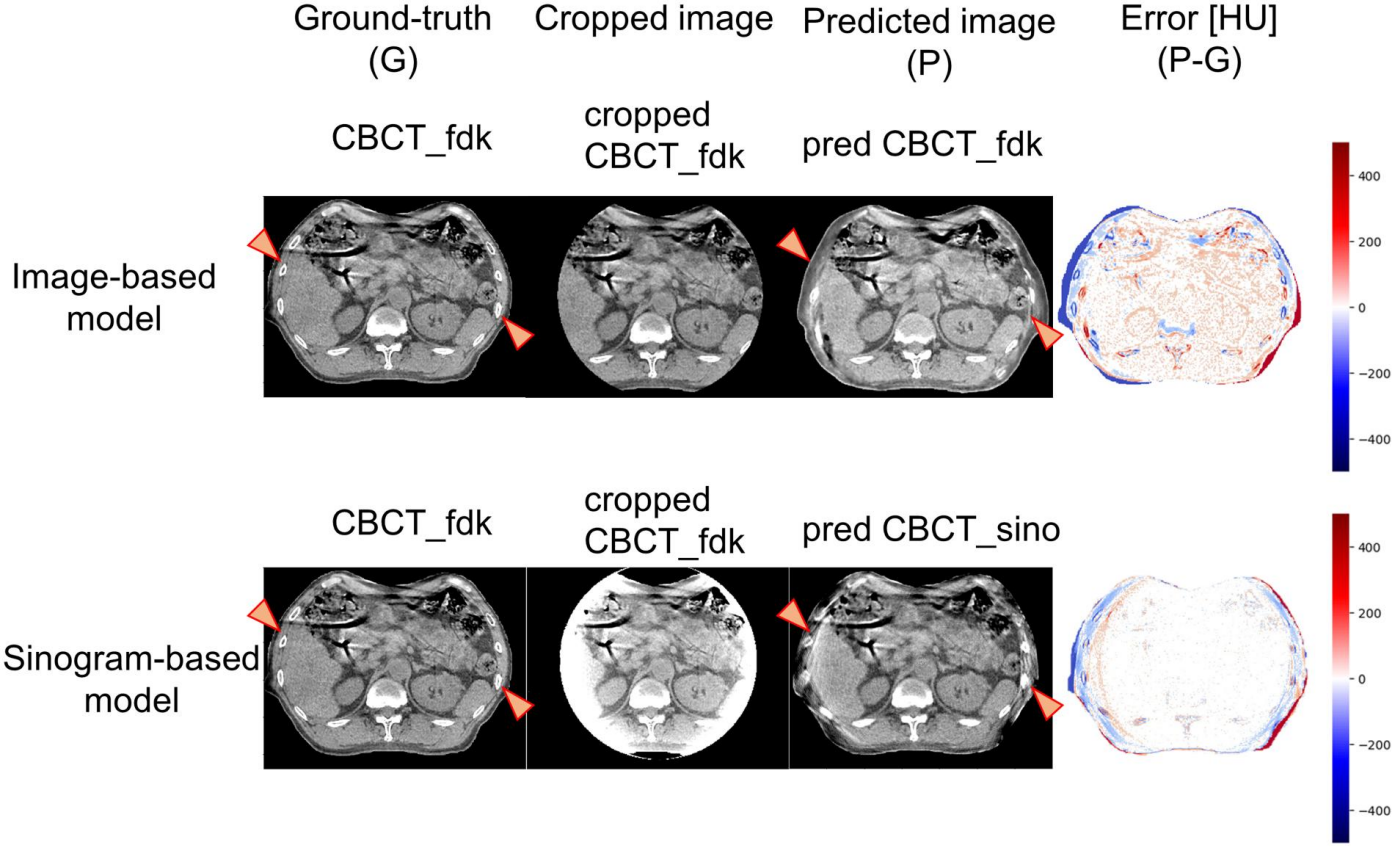
Representative patient images for the CBCT external test datasets, including ground-truth, cropped image, predicted image, and the difference (predicted image − ground-truth) for both image- and sinogram-based models. The window width was set to 400 HU, with the window level set to 0 HU. In the heatmap, the maximum value for red was set to 500 HU, and the maximum value for blue was set to −500 HU.

Figure 5 presents the results of the image and difference heat maps for the predicted images and the corresponding ground truth for the CBCT external test datasets. The figure includes representative patient data, including HU value histograms, and presents a comparison of the predicted CBCT_fdk, cropped CBCT_fdk, and CBCTfdk. Discrepancies in HU values at locations with bony structures were highly pronounced compared with those when fCT was used. The sinogram-based model demonstrated superior accuracy compared with the image-based model in both CT and CBCT evaluations.

## Discussion

The results of this study demonstrated the effectiveness of restoring missing regions from limited FOV in pCT and CBCT for radiation therapy. The sinogram-based model consistently outperformed the image-based model across all evaluation metrics. This model accurately predicted missing regions from the limited FOV, accommodating patient-specific irregularities while maintaining continuity of body contour with the FOV boundaries in the cropped image. Additionally, the model avoids inaccuracies in bone positioning that were observed with the image-based model. These findings indicate that the sinogram-based model is superior for reconstructing accurate and complete CBCT images. In practical clinical terms, the interpretation of our quantitative metrics underscores this superiority. For instance, in the internal pCT tests, the sinogram-based model achieved a median MAE of 16.91 HU compared to 101.73 HU for the image-based model. In radiotherapy, CT value accuracy is directly tied to electron density and subsequent dose calculation. A deviation of ∼100 HU could lead to significant dosimetric errors, whereas an error of ∼16 HU falls within a much more acceptable clinical tolerance.^15^ Furthermore, situating these findings within clinical workflows, the ability to accurately restore anatomical context outside the FOV, such as the exact position of ribs and the external body contour, allows radiologists and oncologists to perform accurate dose accumulations without the need for additional, higher-dose half-fan scans. This can streamline clinical workflows and reduce patient radiation burden.

The sinogram-based model demonstrated superior performance in restoring missing regions from a limited FOV compared with the image-based model because of the continuity of information in the sinogram, which is beneficial for DL models.^7–12^ In this study, a sinogram was generated from DRR. Consequently, the continuity of image information in sinograms likely enhanced the accuracy of predicting missing body contours, resulting in superior performance compared with image-based models (Figures 3 and 4). Although the sinogram-based model exhibited considerable potential in fCT evaluations, it faced challenges when applied to CBCT_fdk (Figure 5). This discrepancy could be attributed to differences in image quality between CT and CBCT. In this study, the sinogram-based model trained on CT was applied to CBCT data. To enhance accuracy, future studies could involve developing models specifically customized for CBCT or refining CBCT image quality to resemble CT images.^16–20^

In this study, image-based models did not accurately restore missing regions from outside the limited FOV because of the lack of continuous body contour information caused by missing image data. Huang et al indicated that their method ensures the reliability of structures within the FOV but faces limitations regarding structures outside the limited FOV.^7^ These structures rely heavily on the acquired images, and erroneous structures beyond the FOV may not be corrected.^7^ Kim et al reported that the image-based model using globally and locally consistent image completion with a GAN achieved high similarity, resulting in improved RMSE and SSIM.^8^ However, visual evaluation revealed excessive blurring outside the FOV. For models that restore the image itself, information continuity is lost when the circular area is hollowed out, limiting the ability of the model to provide predictive cues. Even with pix2pix, the limited information continuity in image-based models hinders accurate recovery of body contours. Therefore, the model could not accurately predict patient-specific body contours and rib positions. Thus, models that are more likely to complement the continuity of information, such as sinogram-based models, are more important for restoring missing parts than image-based models.

While our study focuses on model performance, deploying DL in critical fields such as radiation therapy requires careful consideration of ethical, privacy, and cybersecurity issues. In clinical practice, the reliability and explainability of DL models are essential to ensure patient safety and build trust among clinicians. Using a pre-trained model that contains only numerical weights may pose few direct risks. However, to maintain or improve model accuracy, continuous retraining (remodeling) using new clinical data, such as sensitive pCT and CBCT images, is often necessary. If a DL system automatically accesses and extracts patient data from a hospital’s internal network—which increasingly operates as an Internet of Things (IoT) system—it creates significant vulnerabilities regarding cybersecurity and patient privacy. Furthermore, the unplanned use of patient data for secondary training without proper consent raises ethical concerns. Similarly, when sharing the model with external institutions to retrain it using their local data, strict data management is required to prevent privacy breaches. Therefore, managing and sharing this type of healthcare data demands strong security protocols. As highlighted in recent literature, utilizing advanced cyber threat detection models is crucial for protecting IoT hospital imaging networks against cyberattacks.^21^ Future clinical applications and distribution of our proposed model must carefully address these ethical guidelines, ensure explainability, and implement robust cybersecurity measures to protect patient privacy.

This study has several limitations. First, the CT and CBCT images used for evaluation were not obtained under typical clinical conditions. For standardization, these images were converted to DRRs and subsequently processed using the FDK method. Consequently, adapting the model developed in this study to clinical CBCT scenarios could be challenging because of discrepancies between the experimental conditions and real-world clinical settings. Second, the study was limited to evaluations of abdominal images. Since the training, validation, and testing datasets were all derived from abdominal scans, the applicability of the proposed method to other anatomical regions remains undetermined.

## Conclusions

The DL model developed in this study successfully restored missing regions from a limited FOV, with the sinogram-based model demonstrating significantly better performance than the image-based model. The sinogram-based approach achieved lower MAE and RMSE values, and higher SSIM values, indicating greater accuracy in reconstructing anatomical structures. These findings suggest that the sinogram-based model offers a more reliable method for improving the quality of CBCT images in situations where full anatomical information is not available within the FOV.

## Data Availability

The data are not publicly available due to privacy or ethical restrictions.
